# Lost in mTORC1-related translation limits healing, repair and regeneration in mammals

**DOI:** 10.3389/fcell.2023.1294934

**Published:** 2023-11-23

**Authors:** José M. Izquierdo

**Affiliations:** Centro de Biología Molecular Severo Ochoa (CBMSO), Consejo Superior de Investigaciones Científicas, Universidad Autónoma de Madrid (CSIC/UAM), Madrid, Spain

**Keywords:** mTORC1, *de novo* translation, wound healing, repair, regeneration, regenerative medicine

## 1 Introduction

Wound healing is a coordinated process that can be divided into three general phases: inflammatory processes, tissue formation and tissue remodeling. The molecular and cellular events involved in healing, repair and regeneration are still poorly understood, and current therapies are limited. As a result, defective wound healing affects millions of people worldwide every year. Beyond the current wound healing dogma, new mediators and regulatory nodes are continuously being discovered, opening new therapeutic avenues (Wilkinson and Hardman, 2020).

There are several common features of healing, repair and regeneration between animals and humans, such as the processes involved in re-epithelialization, extracellular matrix degradation and remodeling, and regulation of immune response genes, etc. However, there are also many differences; for example, regeneration can occur by blastema formation (e.g., limb regeneration in salamanders, tail regeneration in tadpoles), without proliferation (e.g., morphallaxis in Hydra), or by epimorphic regeneration and morphallaxis (e.g., annelids and planarians). It is nevertheless unclear whether regeneration involves similar molecular mechanisms that are conserved in distantly related taxa, or whether the ability to regenerate damaged tissues is a trait that has evolved or devolved in mammals (lismaa et al., 2018).

The aquatic salamander axolotl (*Ambystoma mexicanum*) is the poster child for limb regeneration, as it has a remarkable ability to heal wounds without scarring and to regenerate most of its structures. Axolotls thus provide us with an opportunity to learn about healing and regeneration in complex organisms. A better understanding of what drives these processes in the axolotl could lead to the development of therapies to improve tissue repair after injury and treat diseases (lismaa et al., 2018).

In their recent Nature article, Zhulyn et al. (Zhulyn et al., 2023) identify mammalian target of rapamycin complex 1 (mTORC1)-mediated *de novo* protein synthesis as a primary hub for healing and regeneration in the axolotl. These findings advance our understanding of wound healing and repair, and offer new opportunities to address essential aspects of organismal repair and regeneration in the context of regenerative medicine.

## 2 mTORC1-linked translatome reprogramming improves healing, repair and regeneration in axolotl

**FIGURE. 1:**
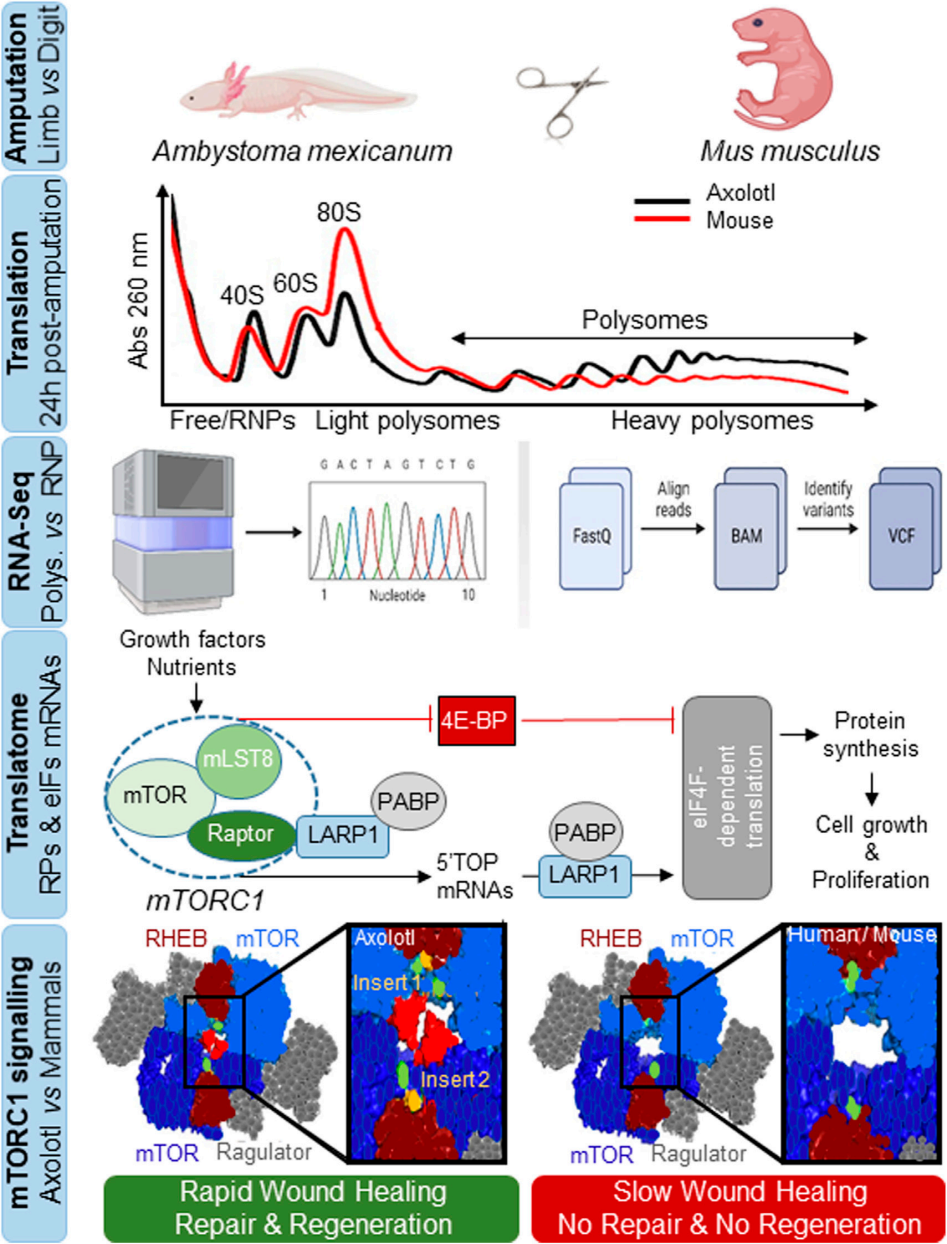
Rapid protein synthesis and mTOR-associated translatome remodeling triggers wound healing, tissue repair and organ regeneration in axolotl. Identification of translationally-active mRNAs by subcellular fractionation and sequencing analysis of light and heavy polysomal fractions against free RNAs or ribonucleoprotein particles (RNPs), involving remodeling of a translatome associated with the overrepresentation of immune component mRNAs, ribosomal proteins (RPs) and eukaryotic translation initiation factors (eIFs). RPs and eIFs mRNAs are known as TOPs mRNAs as they are characterized by a short 5′untranslatable region and a tract oligopyrimidine-rich sequence (5′TOP). They are preferentially translated through the mTORC1 signaling pathway in response to growth factors, nutrients, hormones, cytokines or stress through the collaboration of La-related protein 1 (LARP1) and poly(A)-binding protein (PABP) proteins in a manner dependent on the translation initiation factor eIF4F (eIF4E + eIF4G), or are inhibited through mTORC1-dependent phosphorylation of eIF4E-binding proteins (4E-BP), to regulate protein synthesis and promote cell growth and proliferation. Shown is the evolutionary divergence of the AxmTOR with two insertions (insert 1 and 2) in its primary amino acid sequence *versus* mouse and human mTORC1. The AxmTOR heterodimer is more efficient for subcellular localization and mTORC1 hypersensitization during translation initiation. RHEB and Ragulator are protein regulators that modulate mTOR function. AxmTOR drives rapid wound healing, tissue repair and regeneration in axolotl.

The recent study by Zhulyn et al. (2023) first examined whether beyond changes in transcriptional dynamics during wound healing (Bryant et al., 2017), additional mechanisms are operative in axolotl that confer the almost unexpected ability to drive wound responses in as little as 12 h, as opposed to non-regenerative species such as mammals in which this response is muted and involves extensive scarring. The observation that axolotl genes are 13-fold longer than human equivalents (Nowoshilow et al., 2018), suggest the existence of other regulatory mechanisms that might accelerate healing and regeneration (Figure 1). It is likely for this reason that the authors focused on the regulation and modulation of translation rates (*de novo* protein synthesis), which they studied at the amputation site in axolotl and in digit-amputated neonatal mice (as a non-regenerating control) using polysome sequencing to identify *pre-existing* mRNA transcripts bound to ribosomes (Figure 1). Results showed much higher rates of protein synthesis at the amputation site in axolotl, indicating that it “stockpiles” mRNAs.

By analyzing the mRNAs translated in response to limb injury using sequencing of the free ribonucleoprotein fractions and light and heavy polysomal fractions, the authors identified many showing at least a 2-fold increase in heavy polysomes, indicating increased translation. Some of these mRNAs also exhibited a change in translational efficiency without transcriptional changes, corresponding to pre-existing damage-responsive transcripts not synthesized *de novo*. When the mRNAs were compared against a single-cell RNA-seq dataset, one-half were related to epidermal cells while more than one-third of the transcriptionally-activated mRNAs corresponded to immune cells associated with immune responses, and a decrease was found in dedifferentiation-related transcripts linked to tissue-specific development (Gerber et al., 2018). These results indicated that translationally and transcriptionally regulated mRNAs impact different biological processes in response to amputation; for example, many of the translationally-regulated mRNAs synthesized ribosomal proteins and translation initiation factors (Figure 1). This latter finding drew the attention of the authors to mTOR, which binds to the 5’ terminal oligopyrimidine-rich sequence motifs of these genes (Avni et al., 1994). Indeed, they identified 101 orthologs in axolotl as targets of the mammalian mTOR pathway, allowing them to posit that activated mTOR contributes to translational/translatome remodeling after injury. Many of the translationally overexpressed mRNAs were also redox stress regulators, or were involved in inflammation, phagocytosis, regulation of epithelial-to-mesenchymal transition, cell proliferation and regulation of early wound healing, regulated through mTOR.

mTOR kinase is the catalytic subunit of both the mTORC1 and mTORC2 complexes (Liu and Sabatini, 2020) and plays an essential and evolutionarily conserved role in integrating responses to environmental challenges to regulate cell growth, proliferation and metabolism (Figure 1). The authors found that mTORC1 signaling was active in axolotl during the entire process from injury to healing. Importantly, inhibition of mTOR with INK128 (MLN012) (Hsieh et al., 2012) negatively affects regeneration, and treatment with the small molecule translation inhibitor 4EGl1 (Moerke et al., 2007) reproduced these findings. These observations contrasted with those in mice, where neither increased protein synthesis nor increased mTORC1 activity occurred after amputation. Furthermore, the authors found that mTOR activation was involved in key aspects of both early- and long-term tissue regeneration in axolotl, pointing to its importance in maintaining tissue integrity.

The authors then looked for differences in mTORC1 signaling between axolotl and mice, finding that while the components of this pathway were highly conserved (∼88% amino acid identity with mouse and human orthologs), differences were evident in the intracellular distribution of mTORC1 within tissues: mTORC1 was localized cytoplasmically in mice, whereas in axolotl a pool of mTORC1 was constitutively localized in lysosomes, which might allow its rapid mobilization (Figure 1).

To search for an explanation for the different global and specific translational behavior between axolotl and mouse, the authors looked at the primary amino acid sequence in mTOR across more than 100 metazoan species (mostly vertebrates) including 9 species of urodele amphibians. Analysis revealed that although axolotl mTOR (AxmTOR) is highly conserved, it contained two highly conserved peptides or inserts in the M-HEAT region within amphibian orders: insert 1 has 8 residues within the M-HEAT region critical for RHEB-mediated activation and is conserved in three orders of amphibians; insert 2 contained 20 residues within the M-HEAT domain and was exclusive to urodele amphibians. Moreover, insert 2 expanded the M-HEAT and N-HEAT interface between AxmTOR proteins in the AxmTOR dimer, suggesting a role in dimerization (Figure 1).

To demonstrate the functional relevance of these insertions, the authors genetically modified HEK293 cells to express an ‘axolotized’ version of mTOR. Upon nutrient deprivation, they found that AxmTOR localization in lysosomes was less sensitive to fasting than was mTOR in wild-type HEK293, and that a pool of AxmTOR persisted in lysosomes. They tested whether the insertions affected mTOR function by quantifying mTOR activity under conditions of nutrient and amino acid stimulation, as nutrient-mediated signaling has been suggested to play an important role in tissue regeneration (Ma et al., 2021). Results showed that the chimeric AxmTOR-HEK293 cells were more sensitive to changes in nutrient concentration with an increase in activity associated with AxmTOR and its substrates. The authors hypothesize that the AxmTOR insertions hypersensitize mTOR activity, increasing its ability to generate more efficient dimer, resulting in increased catalytic activity (Figure 1).

## 3 Discussion

During organogenesis, mTOR is pivotal in regulating physiological cell growth and proliferation. In adult organisms, mTOR is involved in several tissue/organ regeneration processes, for example, the regeneration of appendages such as limbs, tails or fins, which involves the proliferation and remodeling of numerous cell types. In the mouse, regeneration processes have been shown to use the same steps of wound closure, blastema configuration, cell proliferation and differentiation. In mammals, however, regeneration is limited to epithelial and connective tissues (lismaa et al., 2018). Zhulyn et al. (2023) now show that regeneration in the salamander is mediated by a specific structural adaptation of mTOR (hypersensitive version), suggesting that small structural changes in key metabolic regulators might distinguish between regenerating and non-regenerating animals.

A fundamental activity of our body is to build new cells and destroy damaged ones. This delicate balance is modulated by mTOR (Liu and Sabatini, 2020), which plays a central role in the metabolism-driven mechanisms that underpin many human diseases by acting prominently on signaling pathways that govern essential cellular activities including growth, proliferation, motility, energy consumption and survival. mTOR signaling is activated when cells detect one of the following: i) amino acids in circulation (after ingestion of protein), ii) growth factors such as insulin (triggered after carbohydrate ingestion), iii) energy (either glucose or fat) and iv) DNA damage. Activated mTOR: i) inhibits autophagy, or recycling of damaged parts of the cell; ii) kick-starts protein and DNA/RNA synthesis; iii) inhibits apoptosis of damaged cells; and iv) stimulates glucose metabolism (Liu and Sabatini, 2020).

Despite the importance of the findings (Zhulyn et al., 2023), it remains unclear whether AxmTOR is directly responsible for regeneration or whether it is linked to other conditions that create a more favorable environment for this to occur. For instance, we do not know whether any hierarchy exists among the hundreds of mRNAs that are translated after injury and that could be directly responsible for the events leading to wound healing and regeneration. In addition, the ability of wound healing and regeneration in genetically-modified mouse and salamander models remains to be addressed.

Notwithstanding these unresolved issues, the findings of Zhulyn et al. (2023) raise several interesting questions: i) what effects do caloric restriction or drugs such as rapamycin, metformin or resveratrol, which regulate mTOR activity, have on tissue repair and regeneration in axolotl?; ii) could the repair and regeneration programs be verified *in vivo* in gain- and loss-of-function animal models such as “axolotlized mice” and “mice-like axolotls”?; iii) can the same biological program of gene remodeling associated with elevated *de novo* cellular translation be established in the absence of healing, injury or organ repair/regeneration to treat pathologies such as cancer or inflammatory diseases such as obesity, diabetes and myocardial infarct; iv) could hypersensitized mTOR be reproduced by generating new specific drugs and/or nutrients (e.g., leucine) that increase its selective translational activity without unwanted side effects?; and v) could personalized medicine use cell lines expressing hypersensitized mTOR to trigger early wound healing and stimulate tissue regeneration in situations such as injuries from accidents, burns from fires, ulcerations associated with diabetes and other human pathologies with anomalous healing?

In summary, Zhulyn et al. (2023) present an evolutionary divergent explanation of why some species quickly heal wounds and regenerate organs whereas mammals cannot. The key regulatory protein could be AxmTOR, which drives the rapid increase in *de novo* protein production in response to injury. These findings have far-reaching implications for healing in mammals, and highlight future cell therapy strategies to advance regenerative medicine.

## References

[B1] Avni D. Shama S. Loreni F. Meyuhas O. (1994). Vertebrate mRNAs with a 5’-terminal pyrimidine tract are candidates for translational repression in quiescent cells: characterization of the translational cis-regulatory element. Mol. Cell. Biol. 14, 3822–3833. 10.1128/mcb.14.6.3822 8196625 PMC358749

[B2] Bryant D. M. Johnson K. DiTommaso T. Tickle T. Couger M. B. Payzin-Dogru D. (2017). A tissue-mapped axolotl *de novo* transcriptome enables identification of limb regeneration factors. Cell Rep. 18, 762–776. 10.1016/j.celrep.2016.12.063 28099853 PMC5419050

[B3] Gerber T. Murawala P. Knapp D. Masselink W. Schuez M. Hermann S. (2018). Single-cell analysis uncovers convergence of cell identities during axolotl limb regeneration. Science 362, eaaq0681. 10.1126/science.aaq0681 30262634 PMC6669047

[B4] Hsieh A. C. Liu Y. Edlind M. P. Ingolia N. T. Janes M. R. Sher A. (2012). The translational landscape of mTOR signalling steers cancer initiation and metastasis. Nature 485, 55–61. 10.1038/nature10912 22367541 PMC3663483

[B5] Iismaa S. E. Kaidonis X. Nicks A. M. Bogush N. Kikuchi K. Naqvi N. (2018). Comparative regenerative mechanisms across different mammalian tissues. NPJ Regen. Med. 3, 6. 10.1038/s41536-018-0044-5 29507774 PMC5824955

[B6] Liu G. Sabatini D. M. (2020). mTOR at the nexus of nutrition, growth, ageing and disease. Nat. Rev. Mol. Cell. Biol. 21, 183–203. 10.1038/s41580-019-0199-y 31937935 PMC7102936

[B7] Ma C. Teng L. Lin G. Guo B. Zhuo R. Qian X. (2021). L-leucine promotes axonal outgrowth and regeneration via mTOR activation. FASEB J. 35, e21526. 10.1096/fj.202001798RR 33813773

[B8] Moerke N. J. Aktas H. Chen H. Cantel S. Reibarkh M. Y. Fahmy A. (2007). Small-molecule inhibition of the interaction between the translation initiation factors eIF4E and eIF4G. Cell 128, 257–267. 10.1016/j.cell.2006.11.046 17254965

[B9] Nowoshilow S. Schloissnig S. Fei J. F. Dahl A. Pang A. W. C. Pippel M. (2018). The axolotl genome and the evolution of key tissue formation regulators. Nature 554, 50–55. 10.1038/nature25458 29364872

[B10] Wilkinson H. N. Hardman M. J. (2020). Wound healing: cellular mechanisms and pathological outcomes. Open Biol. 10, 200223. 10.1098/rsob.200223 32993416 PMC7536089

[B11] Zhulyn O. Rosenblatt H. D. Shokat L. Dai S. Kuzuoglu-Öztürk D. Zhang Z. (2023). Evolutionarily divergent mTOR remodels translatome for tissue regeneration. Nature 620, 163–171. 10.1038/s41586-023-06365-1 37495694 PMC11181899

